# Raising Hopes, Facing Challenges: Understanding Parental Well-Being in the Midst of Autism in Saudi Arabia

**DOI:** 10.3390/bs14070531

**Published:** 2024-06-25

**Authors:** Shuliweeh Alenezi, Norah Alsewailem, Bayan A. Almubaddil, Rand Saud Alshaya, Sarah Mohammed A. Alqahtani, Sara A. Aldossari, Shimah Maibed Alsalhi, Ahmed S. Alyahya

**Affiliations:** 1 Department of Psychiatry, College of Medicine, King Saud University, Riyadh 12372, Saudi Arabia; salenizi@ksu.edu.sa (S.A.); norah.alsewailem@gmail.com (N.A.); abdulaziz.bayan.a@gmail.com (B.A.A.); rand.s.alshaya@gmail.com (R.S.A.); sarah.m.a.alqahtani@gmail.com (S.M.A.A.); sara963963s@gmail.com (S.A.A.); shimah.salhi@gmail.com (S.M.A.); 2 Department of Psychiatry, King Saud University Medical City, King Saud University, Riyadh 12372, Saudi Arabia; 3 SABIC Psychological Health Research and Applications Chair (SPHRAC), Department of Psychiatry, College of Medicine, King Saud University, Riyadh 11451, Saudi Arabia; 4 Division of Psychiatry, Department of Medicine, King Abdullah bin Abdulaziz University Hospital, Princess Nourah bint Abdulrahman University, Riyadh 11564, Saudi Arabia

**Keywords:** parents, autism spectrum disorder, anxiety, depression, quality of life

## Abstract

Background: This study aimed to investigate the levels of anxiety, depression, and quality of life among parents of children with autism spectrum disorder. It also compared the difference in these levels between mothers and fathers. Additionally, it quantifies the association between parental anxiety, depression, and quality of life, with various sociodemographic factors. Methods: This analytical, cross-sectional study was carried out between June and December 2022. An online questionnaire was completed by a sample of 394 parents of children with ASD (autism spectrum disorder) residing in Saudi Arabia. Three scales were used to assess depression, anxiety, and quality of life (QoL), respectively: Patient Health Questionnaire 9 (PHQ-9), Generalized Anxiety Disorder 7 (GAD-7), and World Health Organization Quality of Life—Brief Version (WHOQOL-BREF). Results: Most parents (70.8%) were mothers with an average age of 39 years, typically ranging from 30 to 48 years. Parents’ overall quality of life was 57.72 out of 100, indicating moderate satisfaction. Anxiety levels showed that 32% had mild, 17.8% moderate, and 14.7% severe anxiety. Similarly, depression levels revealed that 34.5% had minimal, 32.2% mild, and 18% moderate depression. Higher anxiety and depression scores were linked to a lower perceived QoL (quality of life). Moreover, the socioeconomic status index (SESi) was significantly and positively correlated with higher depression and lower quality of life. Conclusions: Autism Spectrum Disorder imposes a strain on parents of the autistic child. The responsibilities linked to the disability amplify the occurrence of depression and anxiety among parents, leading to a diminished quality of life.

## 1. Introduction

Autism spectrum disorder (ASD) is a complicated neurodevelopmental disorder characterized by persistent difficulties with social communication, restricted interests, and repetitive behavior [1]. ASD has been reported in all races, ethnicities, and socioeconomic groups. The Centers for Disease Control and Prevention (CDC)’s Autism and Developmental Disabilities Monitoring (ADDM) Network estimates that 1 in every 44 children has ASD [2]. According to a systematic review of the epidemiology of autism in the Gulf countries, the prevalence ranges from 1.4 to 29 per 10,000 persons [3]. However, there are limited data on the national prevalence of ASD in Saudi Arabia, with a few studies citing prevalence rates of 18 per 10,000 and 1 in 167 individuals [4]. Autism is expected to cost an average of SAR 60,000 annually throughout childhood [5]. Parents of children with ASD face significant stress due to various factors, including the costs associated with therapies and specialized education, managing the disruptive behaviors of children with ASD, changes in parents’ social relationships, poor physical health, lack of social support, changes in daily routine, and an insufficient capacity to cope with stress [6]. It has been found that parents of children with ASD are more likely to endure severe psychological distress than parents of typically developing children or even children with other neurodevelopmental disabilities [7,8]. Much work on the potential of developing parental depression and anxiety in parents of children with ASD has been carried out worldwide. Some studies in Saudi Arabia found that depression and anxiety levels in parents of children with ASD were significantly higher than in parents of typically developing children [9,10]. In other Arab countries, caregivers of children with ASD had high levels of stress, depression, and anxiety [8,11], which supports the point of view that parents of children with ASD face more child-rearing stress and psychological discomfort [12]. Some risk factors concerning the child, including ASD symptom severity, comorbidities, and gender (female), were significantly associated with parental psychological distress [13].

Furthermore, parents reported that caring for children with ASD imposed time constraints on their daily lives and routines [14]. Therefore, parents of children with ASD were found to have a lower quality of life across all domains (physical, psychological, social, and environmental) compared to parents of typically developing children [15]. Some parents with a low quality of life took up self-harm and crying as coping mechanisms, which mainly resulted in more complicated emotional challenges for the parents [16]. Multiple sociodemographic factors can impact the parental quality of life, including gender, employment status, living conditions, and education level [17]. Fathers of children with ASD have a better quality of life than mothers, especially regarding the personal stress aspect [18,19]. This is likely because mothers are the primary caregivers, indicating that they typically take on more responsibility for their children’s care than fathers. Nevertheless, working mothers had a considerably higher quality of life than non-working mothers [6,18]. This difference may have resulted from the ability of working mothers to raise their socioeconomic status and, as a result, improve their quality of life [6]. Several factors were found to affect parental stress in Western countries, including gender (female), challenges from the developmental problems of children with ASD, and unsatisfactory levels of support offered to individuals with ASD and their families [19]. However, in Arab countries, the impact of ASD on parents has just been undertaken in clinical research. Nevertheless, a recent systematic review has found a relationship between the severity of ASD, social support, economic status, maternal level of education, financial burden, marital status, parental age, and the child’s gender. Furthermore, the most commonly used coping strategy by parents was religious coping, followed by acceptance, positive reframing, withdrawal social events, denial of the diagnosis, increasing social interactions, and accessing information [20].

These findings, taken together, demonstrated an increase in depression and anxiety and a lower quality of life in parents of children with ASD and revealed a difference between mothers’ and fathers’ psychological impact. As a result, this study sought to fill a gap in the literature by examining the psychological effects of having a child with ASD on parents and their quality of life, while considering potential gender differences among parents of children with ASD in Saudi Arabia.

## 2. Methods

The present study is an analytical, observational, cross-sectional, questionnaire-based study. It was conducted from June to December 2022 at the College of Medicine, King Saud University, Riyadh, Saudi Arabia.

### 2.1. Recruitment

The inclusion criteria included the parents of at least one child diagnosed with ASD living in Saudi Arabia. Moreover, the exclusion criteria included parents of children with ASD residing outside Saudi Arabia and parents of ASD children over the age of 18.

### 2.2. Sample Size

A sample of 394 parents of children with ASD in Saudi Arabia was estimated using the online Raosoft^®^ sample size calculator, with a 5% margin of error and a 95% confidence interval, and adding 50% to the sample size to ensure a reasonable response rate [21]. A convenience sampling technique was used.

### 2.3. Data Collection

The data were collected using an online questionnaire. The questionnaire included a sociodemographic section and was distributed to parents of children with ASD who were registered with autism services via the Charitable Society of Autism Families; autism centers; schools; daycares; and groups such as WhatsApp, Telegram, Twitter, and other social media.

### 2.4. Questionnaires

Three validated Arabic-translated scales were used, PHQ-9, GAD-7, and WHOQOL-BREF, to assess depression, anxiety, and quality of life, respectively.

The Patient Health Questionnaire-9 (PHQ-9) Scale contains nine items, each scored 0–3, which corresponds to “not at all”, “several days”, “more than half of the day”, and “nearly every day”, and provides a 0–27 severity score. The level of depression severity is classified as minimal (1–4), mild (5–9), moderate (10–14), moderately severe (15–19), and severe (20–27) [22,23]. A PHQ-9 score ≥ 10 has a sensitivity of 88% and a specificity of 88% for major depression [22,24]. According to the original source, The PHQ-9 demonstrated an internal reliability of Cronbach’s alpha of 0.89 [24].

The Generalized Anxiety Disorder-7 (GAD-7) Scale has seven items, each scoring 0, 1, 2, and 3, which represent “not at all”, “several days”, “more than half of the day”, and “nearly every day”, respectively, with a 0-to-21 severity score. The severity level was categorized into four groups, namely minimal/no anxiety (0–4), mild (5–9), moderate (10–14), and severe (15–21), with a cut-off score of 10 points [23,25]. This cut-off point was identified to optimize sensitivity (89%) and specificity (82%). GAD-7 has a strong internal consistency of Cronbach alpha, α = 0.9 [25].

The World Health Organization Quality of Life—BREF (WHOQOL-BREF) Scale is an abbreviated assessment tool that assesses the quality of life over four domains (physical health, psychological health, social relationships, and environment). It contains 26 items, each scored from 1 to 5. The scores are then presented on a scale from 0 to 100, with higher scores indicating a higher quality of life [26,27]. Sensitivity was 76.8%, and specificity 63.8%, for a cut-off of ≥60. However, sensitivity was 95%, and specificity was 54.4%, for a cut-off of <60. [28] The WHOQOL-BREF scale had a Cronbach’s alpha coefficient of 0.896 [26].

### 2.5. Data Analysis

Descriptive analysis with the mean ($\bar{x}$) and standard deviation was applied to continuously measured variables, and the median and interquartile range (IQR) were used to describe continuous variables that showed statistical normality assumption violation. The frequencies and percentages were used to describe the categorically measured variables. The statistical normality assumption was tested using the histogram and the Kolmogorov–Smirnov statistical test of statistical normality. The multiple response dichotomies analysis was used to describe the variables measured with more than two option answers. The bivariate Spearman’s (Rho) test of correlation was used to assess the correlation between metric variables. The categorical factor analysis (the nonlinear factor analysis) was used as a dimension reduction technique to estimate the parents’ socioeconomic state index via regressing their sociodemographic, educational, income, and social factors against their latent socioeconomic state, and a summarized standardized socioeconomic factor (SES) score was yielded; this SES factor score was used as an independent predictor variable for other health outcomes and quality-of-life perceptions. The multivariable binary logistic regression analysis was applied to assess what may explain the parents of autistic children’s odds of depression and anxiety measured outcomes, and the association between predictor variables and the analyzed outcomes was expressed as a multivariate-adjusted odds ratio (OR) with their associated 95% confidence intervals (CIs). The multivariable linear regression analysis was also applied to assess what may explain the parents’ mean of the perceived quality of life, and the association between the predictor variables and this outcome was expressed as unstandardized beta coefficients with their associated 95% confidence intervals. The IBM SPSS v21 statistical data analysis program was used, and the alpha significance level was considered at 0.50.

## 3. Results

Three hundred and ninety-four parents of children with ASD who were enrolled electively in the study completed and returned the online questionnaire. The resulting findings from the descriptive analysis of the parents’ sociodemographic characteristics (Table 1) showed that most of the respondents, 70.8%, were females, and 29.2% were males. The mean ± SD age for the sample of parents was equal to 39.3 ± 8.7 years, and their age groups were as follows: 16.8% of them were aged between 20 and 30 years, most of them (45.2%) were aged between 31 and 40 years, 27.9% of them were aged between 41 and 50 years, and the remainder (10.2%) was aged ≥51 years. A few of the respondents, 11.7%, were either divorced or widowed, but most of them, 88.3%, were married. Also, the resulting analysis findings showed that 12.9% of the parents were non-Saudi residing and working within the Kingdom, and 81.1% of respondents were indeed Saudi citizens. Regarding the educational level of participants, 7.1% of them had an elementary education, another 7.9% of them had an intermediate educational level, 26.4% of them had high school, 51.3% had finished their undergraduate level, and the remainder (7.4%) had post-graduate degrees.

Regarding the employment status of the participants, 58.6% of them were unemployed, 11.2% of them worked in the private sector, and 30.2% worked in the governmental sector. The Household Monthly Income (HHI) was calculated in Saudi Arabia Riyals (SAR): 23.1% of the respondents had an HHI of <4000 SAR/month, 32.5% of them had an HHI of between 4000 and 8000 SAR/month, 30.5% of them had an HHI of between 8000 and 15,000 SAR/month, and 14.0% of the sample had a household income of >15,000 SAR/month. Additionally, 18.8% of the parents had previously attended social and psychological support groups for children with ASD’s autism problems. Nonetheless, a number of respondents (22.3%) needed more medical insurance at the time of our research. Furthermore, the data revealed that participants from Central Province constituted a majority share, approaching two-thirds (36.8%), followed by those hailing from Eastern (9.9%) and Northern Provinces (7.0%). The analysis of family size indicated a substantial number of respondents reporting having four or more children, comprising 43.9% of the sample, while only a meager proportion of 6.6% had a single child.

In Table 2, Male children predominated among children with ASD, based on parental reports, accounting for around three-quarters (73.6%) of all cases considered. Of these children with ASD, nearly four out of ten belonged to either the age category between 4 and 6 years or that ≥10 years old. Nearly half (48.5%) were diagnosed with ASD within an age range from three to five years old at diagnosis time. Moderate ASD severity accounted for most cases (61.4%), as per parental reporting; additionally, it was found that approximately a quadrant (25.1) of the children had accompanying medical illnesses and comorbidities based on the analysis conducted regarding the exceptional care arrangements made for their children living with ASD. The analysis revealed that 59.9% of parents favored specialized autism centers to cater to their child’s specific needs. Significantly, only 8.1% marked home-based treatment as their preferred option, contrasting sharply with those who provided no additional therapy or support at all (8.6%). The inclusion criteria for recruitment involved having at least one child with ASD and inquiring if there were other members within the family affected by ASD, where only 6.1% of parents answered having such an occurrence.

The findings of the descriptive analysis of parents’ perceptions of depression and generalized anxiety disorder indicators (PHQ-9 and GAD-7 scales) are presented in Table 3. The results revealed that parents primarily perceived feeling tired and lacking energy ($\bar{x}$ = 1.18) as indicative of depression, followed by having little interest or pleasure in doing things ($\bar{x}$ = 1.12) and feeling down or hopeless ($\bar{x}$ = 1.10). The least perceived depression indicators were feeling bad about oneself ($\bar{x}$ = 0.74), moving or speaking slowly ($\bar{x}$ = 0.66), and thoughts of being better off dead ($\bar{x}$ =0.22). Furthermore, for anxiety perceptions, worrying excessively about different things was seen as the top indicator by parents ($\bar{x}$ = 1.26), along with becoming easily annoyed or irritable ($\bar{x}$ = 1.26). At the pinnacle of the anxiety indicators perceived by parents were restlessness, making it challenging to sit still ($\bar{x}$ = 0.75); experiencing feelings of dread as if facing impending doom; and persistent worrying.

Table 4 shows the physical health indicator that was strongly perceived by parents of children with ASD: their satisfaction with their capacity for work ($\bar{x}$ = 3.48). Additionally, they expressed high levels of satisfaction with their performance of daily activities ($\bar{x}$ = 3.30) and quality sleep ($\bar{x}$ = 3.22). The parents of children with ASD also placed great importance on psychological health indicators, such as self-satisfaction ($\bar{x}$ = 3.73), acceptance of bodily appearance ($\bar{x}$ = 3.54), concentration capabilities ($\bar{x}$ = 3.13), and feeling a sense of significance in life ($\bar{x}$ = 3.31). Among social relationships, the parents of children with ASD highly valued satisfaction with personal relationships ($\bar{x}$ = 3.59). However, satisfaction with sex life ($\bar{x}$ = 3.45) and support from friends ($\bar{x}$ = 3.28) were ranked lower, according to the parents’ perceptions. In terms of satisfaction with their environment, parents of children with ASD identified transport ($\bar{x}$ = 3.52) as the most significant indicator, followed by safety ($\bar{x}$ = 3.39), access to healthcare services ($\bar{x}$ = 3.37), and the conditions of their living place ($\bar{x}$ = 3.34).

Table 5 presents the findings regarding anxiety, depression, and quality of life (QoL) perception levels among parents of children with ASD. An analysis of the data indicates that their satisfaction with QoL is moderate, with an average score of approximately (57.72%). Among all aspects considered, parents are most satisfied with their physical well-being, scoring around (60–61%), while environmental satisfaction levels are lower, at 52.13%. According to the results from the GAD-7 scale, a majority of parents experience mild levels of anxiety, with a median score of 6 out of 21. Nevertheless, when considering cut-off values from previous studies, a significant portion of them experienced varying degrees of anxiety, ranging from minimal (35.5%) to severe (14.7%). Regarding the PHQ-9 scores, a majority of parents exhibit mild depression, with an overall median score of 7 out of 31. Categorizing parental depression based on severity reveals that most fall into the minimal depression category, with a score of 34.5%. concurrently, notable variations were observed across different categories regarding the number of individuals affected. For instance, approximately a third (32.2%) reported experiencing mild symptoms, while only around 10% exhibited severely elevated rates, characterized by significantly depressive or often debilitating symptoms (meaning moderately severe or more).

Table 6 displays the bivariate correlations with the Spearman’s (Rho) test between the measured perceptions of parents of children with ASD. The analysis findings showed that the parents’ general satisfaction with life had correlated positively and significantly with their mean overall perceived quality of life (WHOQOL-BREF) score (rho = 0.676; *p*-value ≤ 0.001); also, the parents’ general satisfaction with health had correlated positively and significantly with their mean overall perceived quality of life (WHOQOL-BREF) score (rho = 0.651; *p*-value ≤ 0.010). Moreover, the subscales of the (WHOQOL-BREF) questionnaire (namely physical health, psychological well-being, social satisfaction, and environmental satisfaction) all correlated significantly and strongly with their overall mean perceived quality of life (WHOQOL-BREF) score: *p*-value ≤ 0.010 each, respectively. The parents’ mean perceived generalized anxiety (GAD7) score correlated negatively and significantly with their mean overall perceived quality of life (WHOQOL-BREF) score (rho = −0.691; *p*-value ≤ 0.010), and the parents’ mean perceived ADL difficulty (GAD_DIFF) due to generalized anxiety had correlated also negatively and significantly with their overall QoL score: rho = −0.636, *p*-value ≤ 0.010. Nonetheless the parents’ mean perceived depression (PHQ9) score correlated significantly and negatively with their perceived QoL score: rho = −0.693; *p* ≤ 0.010. Also, the parents’ mean perceived ADL difficulty (PHQ_DIFF) due to depression correlated significantly and negatively with their overall QoL score: rho = −0.595, *p* ≤ 0.010. The parents’ household socioeconomic status (SES) score correlated significantly and positively but very weakly with their physical health satisfaction subscale score of quality of life: rho = 0.121; *p*-value ≤ 0.050. Also, the parents’ SES score correlated positively and significantly with their satisfaction with their environment subscale score: rho = 0.174, *p*-value ≤ 0.010. In addition, the parents’ mean perceived generalized anxiety (GAD7) score had a weak-to-moderate significant negative correlation with all of the WHO quality-of-life subscale scores (physical, psychological, social, and environmental): *p* ≤ 0.010 each, respectively. In addition, the parents’ mean perceived depression (PHQ9) score loaded significantly and negatively to each of the WHO quality-of-life subscale scores: *p*-value ≤ 0.010. The parents’ mean perceived depression (PHQ9) and anxiety (GAD7) score correlated significantly and positively. Also, the parents’ mean perceived ADL difficulties associated with depression (PHQ_DIFF) and anxiety (GAD_DIFF) also correlated significantly and positively with each other and with the anxiety (GAD7) and depression (PHQ9) mean scores.

Table 7 presents a multivariable linear regression analysis on the WHOQOL-BREF score of parents of children with ASD. It was observed that the mean overall perceived quality of life was not significantly influenced by their sex and age. Nonetheless, a significant and negative correlation was identified between parents’ mean perceived GAD-7 and PHQ-9 scores (*p*-value < 0.001) and their mean overall perceived quality of life. Upon analyzing the data gathered from this study, it became apparent that parents—especially those with higher anxiety and depression scores—reported relatively low levels of perceived quality of life. Another noteworthy result is the positive correlation between the parental household socioeconomic status index score and the overall perception of quality of life. Every point increase on this scale corresponded to an increase in average perceived quality of life, scoring approximately 1.353%. Conversely, families with many children registered lower topmost perceived ratings relating to issues associated with their general well-being. Significant evidence suggests that greater difficulty in completing routine tasks induced by anxiety (*p*-value < 0.001) leads to notably lower scores on the overall perception of quality-of-life scales in parents raising children diagnosed with ASD.

We utilized a multivariable binary logistic regression analysis, presented in Table 8, exploring the relationship between parents’ chances of experiencing moderate-to-high levels of depression against their child’s ASD-related factors and sociodemographic characteristics. The findings revealed that variables such as sex, age, and number of children held no direct influence over parental depressive tendencies. Alternatively, a statistically significant correlation (*p*-value = 0.01) demonstrated that parental socioeconomic status (SESi) positively related to increased probabilities for high-level cases; every individual standard point increment within SESi showed an average rise by a factor of 1.941 concerning potential indications for elevated depressive symptoms among parents. Parents who reported higher scores on perceived GAD-7 had significantly greater chances of experiencing high depression, according to our findings. For every one-point increment in the GAD-7 score scale, there was a noticeable average rise in the likelihood that parents may experience high depression, increasing by as much as 46.2%. According to the study’s results, there was a significant association between higher levels of perceived ADL difficulty among parents of children with ASD and increased odds (2.124 times higher) of experiencing elevated depression. Interestingly, the study found a strong negative association between the mean perceived overall quality of life (WHOQOL-BREF) score and the likelihood of higher depression among parents. For each point increase in this score, on average, there was a corresponding decrease in their chances of experiencing high levels of depression by about 3.52%.

The multivariable binary logistic regression analysis examined the probability of parents of children with ASD having moderate-to-high GAD-7-measured anxiety levels. The results shown in Table 9 indicate that, on average, male parents had significantly higher odds (2.309 times more) of experiencing high anxiety compared to their female counterparts. By establishing a statistically significant positive link between the parental mean perceived depression (PHQ-9) score and their odds of having high anxiety levels, it has been revealed that, for every one-point increase in depression score, on average, the predicted odds of experiencing elevated anxiety increase by a factor of 35.9%. Further exploring this relationship led to the uncovering of a negative association between parents’ overall mean quality of life (WHOQOL-BREF) score and the likelihood of suffering from increased anxiety. An increase of one point on this scale led to decreased chances for high-level experiences with anxiety by about 3.7% on average. High levels of anxiety among parents of children with ASD were significantly associated with more significant perceived ADL difficulties due to anxiety.

## 4. Discussion

With the unique characteristics setting ASD apart from other disabilities, parenting a child with ASD can result in considerable stress for caregivers, owing mainly to the child’s resistance towards change and unfavorable behavior [12]. The present study aimed to examine how anxiety, depression, and quality-of-life considerations affect parents who look after children affected by ASD. It is worth noting that discrepancies in findings between different studies are affected by the differences in assessment tools that are used by different studies [9,11,12], as each follows its individualized standards for evaluation and analysis. Numerous investigations show that parents advocating for children diagnosed with ASD show a discernable proclivity towards experiencing high levels of depressive symptoms, as well as emotional distress. As indicated by the existing literature, there exists a connection between heightened parental psychological distress and the added obligations and time constraints involved in caring for a child with ASD coupled with amplified parenting-related concerns. Studies have shown a positive and significant correlation between the severity of a child’s ASD symptoms and parents’ symptoms. For instance, a study demonstrated that parents of children with more severe ASD symptoms experienced higher levels of anxiety and depression [20]. Furthermore, another study highlighted a significant association between the severity of ASD and the overall quality of life of parents across all domains [15].

Through our investigation, we found that the level of satisfaction among parents was moderate, at 57.72%, indicating an average outcome comparable to the results attained from a corresponding study conducted in Jordan, yielding a rate of 61.39% [6]. Nevertheless, despite the fact that the quality of life for parents of children with ASD was moderate, it was still lower than that of other parents. A number of prior studies identified the social relationship domain as having the highest mean score, while the environment domain has been established as having the lowest [6,29]. These results are consistent with those of our own study. One probable explanation for why there was a high score in social relationships might be due to support from the government and society for the parents of children with ASD in Saudi Arabia. A study conducted in Saudi Arabia found that social support serves as a protective factor for life satisfaction among parents of children with ASD [30]. Considering the factors affecting individuals’ QoL standards highlighted by this study, parents’ gender or age did not play a considerable role, according to our findings; however, comparing the existing literature shows interesting distinctions between mothers’ and fathers’ results. The studies suggest a big gap, with fathers reporting substantially higher quality-of-life levels than mothers [31,32,33]. This finding may suggest that mothers, as primary caregivers of children with ASD, face more stress and challenges in their daily lives, negatively impacting their quality of life. As observed from this study’s results, it can be deduced that there is a significant negative correlation between parental anxiety and depression scores and their overall perceived quality-of-life score, which suggests that the presence of anxiety and depression has adverse effects on parental perceived quality of life. The previous literature corroborates this claim, as a similar trend occurred following a research work carried out in India where there was a negative correlation across all domains of QoL metrics with depression [34]. Additionally, an Egyptian-based study discovered significant links between lower quality-of-life scores experienced by parents and their anxiety [35]. Parents’ quality of life positively and significantly correlated with their socioeconomic status. Similarly, other studies identified parental education level as a protective factor for parental QoL [15,29,36], suggesting that socioeconomic status has a significant role in parents’ overall perceived quality of life and could potentially serve as a protective factor against anxiety and depression. Our results found a significant and negative relationship between the number of children in a household and the parents’ quality of life. An additional investigation supports this claim by pointing out how having more than one child diagnosed with ASD has an unfavorable effect on parents’ QoL [16]. Therefore, it conveys that caring for two or more kids can pose challenges, primarily when having an ASD child. According to our results, most parents experienced mild-to-severe anxiety; this aligns with previous surveys reporting similar outcomes [37]. Children diagnosed with ASD can require intensive care and support from their families, resulting in increased stress levels among parents. Several investigations have investigated this association between high anxiety levels and possible influencing factors, such as the parents’ sex. Interestingly, our results showed a greater incidence of anxiety in fathers than in mothers; this was unexpected since many studies worldwide have shown that mothers typically shoulder the majority of caregiving responsibilities and consequences [29,31]. A possible explanation for why fathers were found to have higher levels of anxiety might be because, typically, in Saudi Arabia, the father bears the financial responsibility of the family. A study found a strong and significant correlation between financial worries and psychological distress [38]. Nevertheless, one study came up with an opposite conclusion and found no significant link between parent gender and anxiety [39]. An association between anxiety and depression was established in our results. A study conducted in Iraq found a significant positive correlation between scores for anxiety and depression [40].

Our results highlight a significant association between parental quality of life and level of life satisfaction measured using WHOQOL-BREF scores and parental self-rated satisfaction with their general lives, which had also been noted in an earlier study [41]. Moreover, we found evidence that parents’ ADL difficulties due to anxiety were significantly but negatively correlated with the mean overall quality-of-life score. However, unlike previous studies linking increased parental anxiety and depression to families who have more than one child diagnosed with ASD, with three children displaying significantly more heightened stress, we did not see any such correlations in our data analysis. Our results analysis discovered no association between parental anxiety and developmental delays among children with ASD. Nevertheless, a previous study suggested that heightened parental anxiety symptoms are more likely when their ASD children exhibit severe behavioral issues [42]. As for the prevalence of parental depression, our results demonstrate that most parents experienced mild-to-severe depression; such patterns align with reports from comparative research demonstrating depression rates of 65 and 63.7, respectively [31,43]. We found significant links associating higher depression rates with socioeconomic status indexes and increased anxiety levels. In contrast, a recent study found that the socioeconomic status of parents has not been linked to parental depression [31].

The findings from our study reveal that there exists a significant negative relationship between the mean level of parents’ reported depression and their corresponding quality-of-life scores. Even if no direct cause-and-effect relationship is observed with socioeconomic status or parental psychiatric disorders as potential causes of parental depression, the socioeconomic status index, GAD-7 score, and WHOQOL-BREF score can still serve as important indicators of parental depression. Our results showed that depression was not found to be related to parents’ sex or age. As reported in another study, the parent’s or caregiver’s age and gender were not significantly associated with depression; hence, there was no significant difference in depression between male and female parents [10].

This research emphasized the need to provide psychological support to parents of children with ASD. This is crucial since families that are not receiving family-focused support services are more likely to have unmet healthcare needs for their children requiring specialized care [44]. A study in Saudi Arabia revealed that children from families living abroad were diagnosed earlier than those from families residing in Saudi Arabia, possibly due to superior or more accessible diagnostic services compared to other countries [45]. Additionally, major cities in Saudi Arabia offer superior services compared to smaller cities [46]. The parents of children with ASD who have limited access to professional support services, combined with low socioeconomic status, unemployment, and inadequate living conditions, are at a greater risk of having a poorer quality of life [6]. This underscores the importance of incorporating mental health screening and psychological counseling for parents as part of comprehensive care for children with ASD. The primary limitation of this research is that it did not measure PHQ-9, GAD-7, and WHOQOL-BREF scores in parents of children without ASD. In addition, the unequal distribution of participants across Saudi Arabia’s cities is also worth noting.

## 5. Conclusions

The current study findings suggest that parents of children with ASD experienced a relatively moderate quality of life and had significant rates of mild-to-severe anxiety and mild-to-severe depression. Parents’ mean overall perceived quality-of-life score and their anxiety and depression scores were found to be significantly correlated. Moreover, fathers were found to have a higher level of anxiety than mothers. The socioeconomic status index is a critical factor since it is linked to higher depression levels and a lower quality of life. These findings suggest that the current generation of parents of children with ASD is facing a wide range of mental health issues due to the stress and strain associated with caring for a child with ASD.

### 5.1. Limitations

As this is a cross-sectional study, it is prone to certain biases, such as sampling, recall, and response biases. In addition, causality and direction of causality cannot be determined. Also, the assessment tools utilized rely on self-reported administration; thus, they cannot confirm the presence or the absence of the diagnoses of interest. Nevertheless, this study offers insight into the well-being of the parents of ASD children.

### 5.2. Recommendations

It is recommended to replicate our study with larger sample sizes, rigorous study designs, and at the national level. These studies should investigate the long-term effects of psychological outcomes. This would provide a greater understanding of how psychological consequences can manifest over time and how they are associated with significant economic-, social-, and health-related outcomes. In addition, longitudinal studies are needed to assess causality.

## Figures and Tables

**Table 1:** Descriptive analysis of parents of children with ASD’s sociodemographic characteristics and medical history.

	Frequency	Percentage
Gender		
Female	279	70.8
Male	115	29.2
Age (years), mean (SD)		39.3 (8.7)
Age groups		
20–30 years	66	16.8
31–40 years	178	45.2
41–50 years	110	27.9
≥51 years	40	10.2
Marital state		
Widowed/divorced	46	11.7
Married	348	88.3
Nationality		
Non-Saudi	51	12.9
Saudi	343	87.1
Educational level		
Elementary	28	7.1
Intermediate	31	7.9
High school	104	26.4
Undergraduate	202	51.3
Post-graduate	29	7.4
Employment status		
Unemployed	231	58.6
Private sector employed	44	11.2
Governmental sector employed	119	30.2
Households Monthly Income		
<4000 SAR/M	91	23.1
From 4000 to 8000 SAR/M	128	32.5
From 8000 to 15,000 SAR/M	120	30.5
>15,000 SAR/M	55	14
Medical insurance		
No	306	77.7
Yes	88	22.3
Have you ever been diagnosed with any psychological disorders before?		
No	362	91.9
Yes	32	8.1
Have you ever been to psychological or social support groups for parents of autistic children before?		
No	320	81.2
Yes	74	18.8
Residence region		
Eastern Provinces	39	9.9
Western Provinces	102	25.9
Northern Provinces	31	7
Southern Provinces	77	19.5
Central Region + Alqassim	145	36.8
Number of children		
One child	26	6.6
Two children	91	23.1
Three children	104	26.4
≥4 children	173	43.9

**Table 2:** Descriptive analysis of the children with ASD’s sociodemographic and disease-related characteristics.

	Frequency	Percentage
Gender of children with ASD		
Female	104	26.4
Male	290	73.6
Age of the autistic child		
<3 years	5	1.3
4–6 years	147	37.3
7–9 years	95	24.1
≥10 years	147	37.3
Age diagnosed with autism		
Less than 3 years old	174	44.2
3–5 years old	191	48.5
6–8 years old	29	7.4
Child’s severity of autism rating		
Mild	115	29.2
Moderate	242	61.4
Severe	37	9.4
Does the child have any diseases or other developmental disorders?		
No	295	74.9
Yes	99	25.1
Type of developmental disorder, *n* = 96		
Cognitive problems and mental retardation	27	28.1
Behavioral and ADDH	31	32.3
Sensorimotor deficits	21	21.9
Visual Impairment	6	6.2
Hearing and speech difficulties	27	28.1
Other comorbidity (asthma, convulsions, and GI problems)	43	44.8
Does your child receive any special care?		
He is cared for at home	32	8.1
He doesn’t get care, but he goes to school	37	9.4
He doesn’t receive any special care	34	8.6
He receives care at a private school	55	14.0
Receives care in a specialized center	236	59.9
If child is not receiving special care, why? *n* = 107		
Other reasons	37	34.6
Location (lack of near schools or centers)	28	26.2
Cost (high cost of treatment)	46	43.0
Quality (poor quality of available services)	19	17.8
Services (lack of necessary services)	23	21.5
Order of children with ASD in the family		
The first child	139	35.3
The middle child	132	33.5
The last child	123	31.2
Do you have other children with ASD?		
No	370	93.9
Yes	24	6.1

**Table 3:** Descriptive analysis of the parents’ perceptions of depression and anxiety.

	Mean (SD)	Median	IQR
**Indicators of Depression (PHQ9)**			
PHQ_1 Little interest or pleasure in doing things	1.12 (1.04)	1.00	2.00
PHQ_2 Feeling down, depressed, or hopeless	1.10 (0.95)	1.00	2.00
PHQ_3 Trouble falling or staying asleep, or sleeping too much	1.06 (1.06)	1	2.00
PHQ_4 Feeling tired or having little energy	1.18 (1.00)	1	2.00
PHQ_5 Poor appetite or overeating	0.92 (1.02)	1	1.00
PHQ_6 Feeling bad about yourself—or that you are a failure or have let yourself or your family down	0.74 (1.04)	0	1.00
PHQ_7 Trouble concentrating on things, such as reading the newspaper or watching television	0.83 (1.01)	0	1.00
PHQ_8 Moving or speaking so slowly that other people could have noticed. Or the opposite—being so fidgety or restless that you have been moving around a lot more than usual	0.66 (0.95)	0	1.00
PHQ_9 Thoughts that you would be better off dead or of hurting yourself in some way	0.22 (0.60)	0	0.00
PHQ_DIFF1 Perceived ADL difficulty from anxiety	0.92 (0.90)	1	1.00
**Generalized Anxiety Disorder indicators**			0.00
GAD_1 Feeling nervous, anxious or on edge	1.22 (1.01)	1	1.00
GAD_2 Not being able to stop or control worrying	1.01 (1.00)	1	1.00
GAD_3 Worrying too much about different things	1.26 (1.02)	1	1.00
GAD_4 Trouble relaxing	1.12 (1.03)	1	2.00
GAD_5 Being so restless that it is hard to sit still	0.75 (0.96)	0	1.00
GAD_6 Becoming easily annoyed or irritable	1.26 (1.12)	1	2.00
GAD_7 Feeling afraid as if something awful might happen	0.87 (1.10)	0	1.00
GAD_DIFF If you checked off any problems, how difficult have these problems made it for you to do your work, take care of things at home, or get along with other people?	0.90 (0.86)	1	1.00

The indicators of the PHQ9 scale and the GAD7 scale show statistical normality violation (positive/negative skewness) of the medians and inter-quartile ranges, as well as the means and standard deviations.

**Table 4:** Descriptive analysis of the parents’ perceptions of quality of life (QoL).

	Mean (SD)	Rank
**Physical Health**		
QoL_3 To what extent do you feel that (physical) pain prevents you from doing what you need to do?	2.15 (1.10)	6
QoL_4 How much do you need any medical treatment to function in your daily life?	1.90 (1.14)	7
QoL_10 Do you have enough energy for everyday life?	3.10 (1.01)	4
QoL_15 How well are you able to get around?	2.79 (1.06)	5
QoL_16 How satisfied are you with your sleep?	3.22 (1.16)	3
QoL_17 How satisfied are you with your ability to perform your daily living activities?	3.30 (1.12)	2
QoL_18 How satisfied are you with your capacity for work?	3.48 (1.13)	1
**Psychological Health**		
QoL_5 How much do you enjoy life?	2.96 (0.94)	5
QoL_6 To what extent do you feel your life to be meaningful?	3.31 (1.11)	3
QoL_7 How well are you able to concentrate?	3.13 (0.96)	4
QoL_11 Are you able to accept your bodily appearance?	3.54 (1.10)	2
QoL_19 How satisfied are you with yourself?	3.73 (1.14)	1
QoL_26x How often do you have negative feelings such as blue mood, despair, anxiety, depression?	2.85 (1.08)	6
**Social Relationships**		
QoL_20 How satisfied are you with your personal relationships?	3.59 (1.20)	1
QoL_21 How satisfied are you with your sex life?	3.45 (1.20)	2
QoL_22 How satisfied are you with the support you get from your friends?	3.28 (1.19)	3
**Environment**		
QoL_8 How safe do you feel in your daily life?	3.39 (1.05)	2
QoL_9 How healthy is your physical environment?	3.04 (1.06)	6
QoL_12 Have you enough money to meet your needs?	2.60 (0.96)	7
QoL_13 How available to you is the information that you need in your day-to-day life?	3.06 (0.95)	5
QoL_14 To what extent do you have the opportunity for leisure activities?	2.36 (1.00)	8
QoL_23 How satisfied are you with the conditions of your living place?	3.34 (1.25)	4
QoL_24 How satisfied are you with your access to health services?	3.37 (1.24)	3
QoL_25 How satisfied are you with your transport?	3.52 (1.16)	1

Note: In each domain of quality of life (QoL), parents rank their satisfaction from highest to lowest.

**Table 5:** Descriptive analysis of the parents’ overall perceptions of QoL, anxiety, and depression scores.

	Mean (SD)	Median	IQR	Maximum Possible Score
Overall quality of life (WHOQOL-BREF) score	57.72 (18.78)	59.77	26.23	0–100 points
Physical (Phys) QoL score	60.17 (20.62)	60.71	28.57	0–100 points
Psychological (Psyc) QoL score	57.57 (20.30)	58.33	25.00	0–100 points
Social (SOC) QoL score	61.00 (24.73)	66.67	33.33	0–100 points
Environmental (ENV) QoL score	52.13 (19.06)	53.13	28.13	0–100 points
General satisfaction with life	3.70 (1.00)	4.00	1.00	1–5 points
General satisfaction with own health	3.67 (1.17)	4.00	2.00	1–5 points
Generalized anxiety disorder GAD7 score	7.48 (5.94)	6.00	8.00	0–21
Perceived ADL difficulty (GAD_DIFF) due to generalized anxiety	0.90 (0.86)	1.00	1.00	0–3 points
General health depression (PHQ9) scale score	7.79 (6.08)	7.00	9.00	0–27 points
Perceived ADL difficulty from depression	1.00 (0.90)	1.00	1.00	0–3 points

Note: Some of the measured perceptions showed statistical normality assumption violation (skewness), such as the mean and SD; the median and IQR range statistics were also used.

**Table 6:** Bivariate Spearman’s (Rho) correlations test between the parents measured perceptions.

	WHOQoL-BREF	LifeSats	GHS	Phys	Psyc	SOC	ENV	GAD-7	GAD_DIFF	PHQ-9	PHQ_DIFF
Overall Quality of Life BREF HOQoL-BREF) score	1										
General quality of life satisfaction self-rated score (LifeSats)	0.676 **										
General health satisfaction (GHS) score	0.651 **	0.576 **									
Physical (Phys) QoL score	0.889 **	0.609 **	0.644 **								
Psychological (Psyc) QoL score	0.914 **	0.650 **	0.613 **	0.791 **							
Social (SOC) QoL score	0.861 **	0.530 **	0.513 **	0.648 **	0.688 **						
Environmental (ENV) QoL score	0.888 **	0.625 **	0.551 **	0.739 **	0.789 **	0.663 **					
Generalized anxiety (GAD7) disorder score	−0.691 **	−0.564 **	−0.503 **	−0.636 **	−0.682 **	−0.570 **	−0.569 **				
Perceived ADL difficulty (GAD_DIFF) due to generalized anxiety	−0.636 **	−0.564 **	−0.527 **	−0.619 **	−0.584 **	−0.524 **	−0.534 **	0.645 **			
General health depression PHQ9 scale score	−0.695 **	−0.534 **	−0.516 **	−0.672 **	−0.673 **	−0.570 **	−0.554 **	0.809 **	0.655 **		
Perceived ADL difficulty (PHQ_DIFF) due to depression	−0.595 **	−0.489 **	−0.467 **	−0.585 **	−0.556 **	−0.501 **	−0.472 **	0.608 **	0.812 **	0.651 **	
Household’s socioeconomic status (SES) factor score	0.081	0.043	−0.037	0.121 *	0.058	−0.038	0.174 **	−0.036	0.013	−0.017	−0.027

Note: ** Correlation is significant at the 0.01 level (2-tailed). * Correlation is significant at the 0.05 level (2-tailed).

**Table 7:** Multivariable linear regression analysis of parents perceived quality of life (QoL) score.

	Unstandardized Beta Coefficients	95.0% CI for Beta Coefficient		*p*-Value
		Lower Bound	Upper Bound	
(Constant)	79.922	73.751	86.092	<0.001
Sex = male	−1.239	−4.171	1.693	0.406
Age (years)	0.015	−0.155	0.186	0.860
Generalized anxiety disorder GAD7 score	−0.944	−1.310	−0.578	<0.001
General Health Depression PHQ9 scale score	−0.863	−1.223	−0.502	<0.001
Household socioeconomic status (SeS) factor score	1.353	0.116	2.589	0.032
Number of children in the family	−1.652	−3.082	−0.223	0.024
Perceived ADL difficulty due to generalized anxiety	−5.804	−7.760	−3.848	<0.001

DV = WHOQOL-BREF score. Model overall significance, f(7, 386) = 75.80, *p*-value < 0.001. Model R = 0.761; adjusted R-squared = 0.571.

**Table 8:** Multivariable binary logistic regression analysis of the parents of children with ASD odds of depression.

	Multivariate Adjusted Odds Ratio	95% CI for OR		*p*-Value
		Lower	Upper	
Sex = male	1.006	0.423	2.393	0.989
Age (years)	0.968	0.920	1.019	0.210
Number of children in the family	1.232	0.803	1.890	0.340
Having a child with a developmental disorder/disability	0.697	0.297	1.634	0.406
Household socioeconomic status (SeS) Factor score	1.941	1.306	2.885	0.001
Generalized Anxiety Disorder GAD-7 score	1.462	1.316	1.624	<0.001
Perceived ADL difficulties due to perceived depression score	2.124	1.332	3.389	0.002
Overall mean perceived quality of life (QoL) score	0.965	0.939	0.991	0.009
Nationality = Saudi	0.407	0.143	1.160	0.093
Constant	0.298			0.384

DV = high PHQ score (moderate-to-high depression, no/yes).

**Table 9:** Multivariable binary logistic regression analysis of the parents of children with ASD odds of high anxiety.

	Multivariate Adjusted Odds Ratio	95% CI for OR		*p*-Value
		Lower	Upper	
Sex = male	2.309	1.003	5.313	0.049
Age (years)	1.004	0.958	1.051	0.881
Number of children in the family	0.679	0.457	1.010	0.056
Having an autistic child with developmental disability types	2.098	0.966	4.558	0.061
General health depression PHQ9 scale score	1.359	1.237	1.493	<0.001
Households socioeconomic (SES) status factor score	0.786	0.563	1.098	0.158
Overall mean perceived quality of life (QoL) score	0.963	0.934	0.992	0.013
Perceived ADL difficulty due to generalized anxiety	1.939	1.156	3.254	0.012
Self-Rated general life satisfaction score	0.586	0.362	0.948	0.029
Self-Rated general health satisfaction score	1.357	0.932	1.975	0.111
Constant	0.309			0.417

DV = high GAD7 score (moderate-to-high anxiety, no/yes).

## Data Availability

All the data for this study will be made available upon reasonable request.
